# 土壤中石油烃检测的前处理新方法:硅胶脱水-环己烷提取

**DOI:** 10.3724/SP.J.1123.2023.04019

**Published:** 2023-09-08

**Authors:** Jian QU, Yuwen NI, Haoran YU, Hongxu TIAN, Longxing WANG, Jiping CHEN

**Affiliations:** 中国科学院分离分析化学重点实验室, 中国科学院大连化学物理研究所, 辽宁 大连 116023; CAS Key Laboratory of Separation Science for Analytical Chemistry, Dalian Institute of Chemical Physics, Chinese Academy of Sciences, Dalian 116023, China

**Keywords:** 样品前处理, 气相色谱, 硅胶, 石油烃, 土壤, sample pretreatment, gas chromatography (GC), silica gel, petroleum hydrocarbon, soil

## Abstract

石油作为全球主要能源之一,所带来的环境污染越来越严重。土壤中的石油烃是环保监测的重要组成部分。现有土壤中石油烃检测的主流方法是以丙酮与正己烷的混合溶剂进行提取,旋蒸后利用无水硫酸钠去除残余水分,再用硅镁型吸附剂净化后进行下一步检测。现有方法步骤繁琐,耗时长,需消耗大量有机溶剂,且旋蒸设备不具便携性,难以应用于现场分析。土壤中石油烃提取的关键在于有效去除土壤中的水分。本工作通过对现有标准方法和文献方法的优化和改进,建立了一种新的土壤中石油烃(C10~C40)的前处理方法:硅胶脱水-环己烷提取。其具体步骤是在土壤样品中加入硅胶,经过混合研磨脱除土壤中的水分,然后采用环己烷浸泡提取、Florisil固相小柱净化,即可进行测试。以气相色谱对该方法进行了系统测评,实验结果表明当加入土壤实际含水质量10倍的硅胶、以40 mL环己烷浸泡提取15 min时,能够获得较满意的提取效果。本方法的回收率为74.1%~103.8%,相对标准偏差(RSD)为1.7%~10.1%。本方法简化了样品前处理步骤,减少了有毒溶剂的使用,降低了对环境的污染,提高了工作效率,不仅可用于石油烃的实验室分析,也为石油烃现场快速分析奠定了良好基础。

## Abstract (en)

Oil is a primary source of energy worldwide. However, the use of oil produces large amounts of pollutants, which are detrimental to the environment. The presence of petroleum hydrocarbons in soil is a critical marker of environmental pollution and safety. Rapid on-site detection technology has been broadly used in emergency tracking, offering critical information support for effective reactions to environmental emergencies. Thus, it is expected to play an increasingly critical role in environmental remediation efforts. The current approach for petroleum hydrocarbon detection in soil mainly involves Soxhlet extraction with a combination of solvents, including acetone and *n*-hexane. The samples are then analyzed after rotary evaporation, dehydration with anhydrous sodium sulfate, and purification using a magnesium silica-type adsorbent. Unfortunately, this approach requires sample analysis to be performed in the laboratory, which is tedious and time consuming, and consumes large amounts of solvents. Moreover, the rotary evaporator is not portable. Therefore, this method is not appropriate for the rapid on-site detection of petroleum hydrocarbons. In this study, a rapid on-site detection method based on silica-gel dehydration and cyclohexane extraction was developed for the extraction and pretreatment of petroleum hydrocarbons (C10-C40) in soil. First, an appropriate amount of silica gel was added to the soil, and the mixture was completely ground to eliminate moisture. Next, petroleum hydrocarbons were extracted with 40 mL of cyclohexane, and the extract was cleaned by Florisil solid-phase extraction (SPE) column elution. Finally, the samples were analyzed by gas chromatography (GC) to evaluate the above method. The silica gel exhibited optimal adsorption properties compared with anhydrous sodium sulfate, calcium oxide, and molecular sieves, with recovery of 87.5%. The effects of different soil water content (5%, 10%, and 20%) and silica gel (1, 3, 5, and 10 times the moisture content) dosage on the extraction of petroleum hydrocarbons were investigated. The recoveries of petroleum hydrocarbons increased from 74.0% to 103.8% after 15 min of invasive extraction (relative standard deviation, RSD, <10.1%) when silica gel amounting to 10 times the moisture content was used. Five types of silica gels with different properties were purchased from four manufacturers, and the effects of these silica gels on the dehydration and extraction efficiency of petroleum hydrocarbons in soil were assessed. The results showed that amorphous silica gel led to low recoveries (<60%), spherical silica gel achieved extraction efficiencies of approximately 70%-90%, and alkaline silica gel produced recoveries with poor precision. Therefore, neutral spherical silica gel was used for further experiments. The fingerprints of petroleum hydrocarbons with different carbon numbers are an important reference for identifying pollution sources. Thus, ensuring good recoveries throughout the entire carbon range is necessary to ensure the accuracy of the fingerprint analysis results. The proposed method showed good recoveries for petroleum hydrocarbons of all carbon numbers (75%-101%). The findings above indicate that the developed method could be an efficient means to extract petroleum hydrocarbons from soil for both total quantity and fingerprint analyses. Compared with standard methods, the proposed method requires lower solvent dosages and features simpler processing steps. Another advantage of this method is that it does not require the use of highly toxic halogenated solvents; thus, it does not contribute to environmental pollution. It can be applied to the laboratory analysis of soil petroleum hydrocarbons and coupled with other rapid on-site detection techniques for soil petroleum hydrocarbons, such as infrared spectroscopy and portable GC. However, because it does not include a concentration process, the developed method exhibits relatively low sensitivity. In the future, we plan to develop a simple and flexible on-site sample-concentration system to further improve various indicators of this method.

石油作为全球主要能源之一,在现代社会生产生活中具有举足轻重的地位,而其所带来的环境污染也越来越严重,据统计我国每年约有60万吨石油进入土壤。在石油污染的土壤中,总石油烃(total petroleum hydrocarbon, TPH)是最主要的污染物。总石油烃是一种含有烃类(正构烷烃、支链烷烃、芳烃、脂环烃)及少量其他有机物(硫化物、氮化物、环烷酸类等)的复杂混合物^[1]^。国际上基于碳当量将总石油烃划分为挥发性石油烃(C6~C9)和可萃取石油烃(C10~C40)^[2]^。

石油烃对土壤的污染主要表现为破坏土壤生态系统的结构与功能,改变土壤的水文物理性质,降低土壤的保持力,影响土壤的通透性,造成土壤肥力下降,从而对生长的植物产生毒害作用^[3]^。此外,土壤中的烃类污染物也可通过循环进入水体和大气,造成二次污染,并通过环境暴露和食物链富集进入人体,对环境和人类造成严重危害^[4,5]^。

国务院2016年发布的国发〔2016〕31号《土壤污染防治行动计划》中,将场地土壤的挥发性有机污染物、石油烃等的污染状况调查列为重点任务。土壤样品中石油烃的现场快速筛查是这个任务中重要的组成部分。目前现场筛查方法主要有便携气相色谱法及红外光谱法。土壤样品中石油烃提取的关键在于有效消除土壤中含有的水分对提取的不利影响^[6⇓-8]^。目前常用的方法既有索氏提取、振荡提取、超声萃取等常规技术^[9⇓⇓⇓-13]^,也有超临界流体萃取、加速溶剂萃取、加压流体萃取等新兴技术^[14⇓⇓⇓-18]^。这些方法主要通过使用丙酮与正己烷的混合溶剂来消除提取环节水分的影响,随后再以旋蒸及无水硫酸钠进行样品的浓缩及干燥;也有提前将土壤样品风干后再提取的做法。丙酮是易制毒化学品,含甲基不能用于红外光谱法。另外丙酮干扰样品净化步骤,在固相小柱净化前必须去除。由于旋蒸设备便携性差,混合溶剂提取难以在现场使用。开发一种不使用丙酮及含甲基溶剂的提取技术,同时满足便携式气相色谱及红外光谱现场检测的需要,对于土壤中石油烃的现场检测非常必要。

基于此,本文提出了一种新的土壤中石油烃检测的前处理方法:硅胶脱水-环己烷提取。以气相色谱对此法进行了系统测评,结果表明新方法简化了实验步骤,对土壤中石油烃的提取效果也令人满意,为石油烃现场分析打下了良好基础。

## 1 实验部分

### 1.1 仪器和试剂

A90气相色谱仪(上海仪盟科技),配氢火焰离子化检测器(FID); AEG-120天平(精度0.0001 g,日本SHIMADZU)。

无添加剂的A型柴油标样和B型矿物油标样(德国Dr. Ehrenstorfer);正构烷烃(C10~C40)标准溶液(31000 mg/L,各正构烷烃质量浓度均为1000 mg/L,美国Agilent);丙酮和环己烷(分析纯,麦克林试剂);无水硫酸钠和氧化钙(分析纯,天津大茂);中性球形硅胶(60~100 μm,日本富士);Florisil固相萃取柱(1000 mg/6 mL,百灵威);分子筛(40~60 目,阿拉丁)。

### 1.2 标准溶液的配制

正构烷烃标准溶液用于配制系列标准工作溶液。标准工作溶液中C10~C40共31个正构烷烃的保留时间用于标定不同碳数石油烃的出峰时间范围。用微量注射器移取适量正构烷烃标准溶液,然后用移液枪分别加入适量环己烷稀释,配制成总石油烃质量浓度分别为310、775、1550、3100、6200 mg/L的标准工作液。

混合石油烃溶液:准确移取柴油标样和矿物油标样各0.5 g至100 mL容量瓶中,用正己烷定容至刻度线,摇匀,制得10 g/L的混合石油烃溶液,置于4 ℃冰箱中冷藏保存备用。此混合石油烃溶液用于土壤样品制备。

### 1.3 不同含水量土壤样品的制备

土壤含水率对石油烃的提取效率有着很大的影响,为了模拟不同含水率土壤对本文方法提取效率的影响,配制不同含水率的土壤。

将采回的土壤样品风干,过60~80目筛后,在马弗炉中400 ℃灼烧5 h,去除所含石油烃及有机干扰物质。用移液管取160 mL 10 g/L的混合石油烃溶液,溶于500 mL正戊烷中,制得混合石油烃溶液。将此石油烃溶液与1 kg过筛灼烧后的土壤样品均匀混合搅拌,放置于通风橱中,室温挥发2 h,中间每隔10 min以玻璃棒充分搅拌,溶剂挥发完全后的样品呈松散干燥状,无黏附现象。最终得到含有1600 mg/kg石油烃的土壤样品。在土壤样品中加入相应量的去离子水,拌匀,制得含水率5%、10%、20%的土壤样品,密封,冷藏备用。

### 1.4 样品的前处理方法

准确称取10.0 g土壤样品至研钵中,根据土壤的含水率(以烘干称重法确定),在研钵中加入土壤样品实际含水质量10倍的烘干球形硅胶,经过充分研磨,使土壤呈流沙状、无黏附现象后,将样品完全移入锥形瓶中。在锥形瓶中使用移液管准确加入40.0 mL萃取溶剂。摇匀静置15 min后,取10 mL上清液,使其通过Florisil固相萃取柱进行净化,收集流出液,注入气相色谱仪进行分析。

### 1.5 气相色谱条件

气相色谱柱:UF-1MS(30 m×0.25 mm×0.25 μm,中国科学院大连化学物理研究所现代分析中心);进样口温度:340 ℃;载气:高纯氮气;流速:1.3 mL/min(恒流模式);不分流进样;进样量:1.0 μL;程序升温条件:初始温度60 ℃,以20 ℃/min的速率升温至340 ℃,保持15 min。FID温度340 ℃。

### 1.6 数据分析

根据正构烷烃标准溶液中正癸烷出峰开始时间确定石油烃(C10~C40)的开始时间,正四十烷出峰结束时间确定石油烃(C10~C40)的结束时间。为评价本文方法对不同碳数石油烃的处理效果,根据正构烷烃标准溶液中各正构烷烃的保留时间范围,对C10~C15、C15~C20、C20~C30、C30~C40的回收率情况分别统计并讨论。由于分析石油烃的气相色谱方法最终柱温达340 ℃,在高温段色谱基线抬升明显。因此在定量分析时预先扣除空白基线。为消除水分对定量的影响,所有定量结果都按照含水量进行了干重折算。回收率测定的数据也按照干重进行了折算。土壤含水量按照国标(GB/T 19422.1-2004),以烘干称重法测定。

## 2 结果与讨论

### 2.1 不同碳数段时间划分

不同碳数段石油烃指纹对污染源判断有重要参考意义。在后续的方法学评价中,本文对位于C10~C15、C15~C20、C20~C30、C30~C40时间段的石油烃回收率进行了分别讨论。实际石油烃样品不仅含有正构烷烃,还有大量异构烷烃,其气相色谱图呈驼峰状。这种谱图无法简单将几个单峰加和得到每段的总峰面积,只能对指定时间范围的谱图进行整体积分^[19,20]^。本文根据C10、C15、C20、C30、C40 5种正构烷烃的出峰时间情况划定了几个碳数段的积分时间范围:C10~C15从C10的峰起点到C15的峰落点(≥5.09, <8.65 min); C15~C20从C15的峰落点到C20的峰落点(≥8.65, <11.35 min); C20~C30从C20的峰落点到C30的峰落点(≥11.35, <16.10 min); C30~C40从C30的峰落点到C40的峰落点(≥16.10, <26.90 min)。后续讨论即采用上述时间段划分对石油烃样品进行整体积分,然后进行分析计算。

### 2.2 不同干燥剂对提取回收率的影响

在使用含丙酮的混合溶剂提取、无水硫酸钠除水的通用方法中,必不可少的浓缩步骤需要使用旋蒸设备。而旋蒸仪器高功耗、大体积、易破损的特点使其难以在现场检测中使用。本文尝试使用不同干燥剂(无水硫酸钠、氧化钙、分子筛、硅胶)与土壤研磨脱水后以环己烷直接提取石油烃。考察了20%含水率土壤与不同干燥剂(用量均为10倍含水量)共同研磨后的石油烃提取效率,结果见表1。结果表明当土壤含水量较大时,使用无水硫酸钠除水效果不好,回收率偏低;而分子筛平均回收率较低;氧化钙黏度较大,研磨过程不易操作,各组分间回收率波动较大;相比于无水硫酸钠,使用硅胶作干燥剂时石油烃的总回收率高出20.0%,达到了87.5%。对于不同碳链长度的石油烃,采用硅胶研磨脱水时,除C15~C20稍低于无水硫酸钠外,其他成分的回收率均高于其他干燥剂,且回收效率较其他干燥剂稳定(相对标准偏差(RSD)为4.2%),因此确定使用硅胶作为干燥剂。

**表 1:** 使用不同干燥剂时土壤中石油烃的提取效率(*n*=3)

Desiccant	Recoveries/%(RSDs/%)				
	C10-C15	C15-C20	C20-C30	C30-C40	C10-C40
Sodium sulfate	58.5 (25.5)	83.4 (10.2)	65.5 (11.3)	56.3 (28.5)	67.3 (6.0)
Calcium oxide	40.7 (2.8)	35.9 (15.3)	42.4 (40.2)	66.8 (26.2)	46.2 (19.3)
Molecular sieve (40-60 mesh)	46.9 (33.5)	38.2 (20.1)	31.6 (28.4)	39.6 (11.9)	38.2 (16.6)
Silica gel	85.0 (8.6)	76.8 (4.5)	97.5 (6.7)	86.8 (4.8)	87.5 (4.2)

### 2.3 不同硅胶提取效率的对比

硅胶作为一种具备丰富多孔结构和较高吸附能力的传统干燥剂,具有吸附性能好、机械强度高、价格低廉、对环境无污染等优点,从而在各领域得到广泛应用^[21]^。为了评价不同来源、形状、粒径的硅胶对土壤脱水、石油烃提取效率的影响,分别购买了4个厂家不同性质的硅胶5种,考察了硅胶的性质对土壤中石油烃脱水及提取效率的影响。硅胶的物化性质见表2。使用5种不同硅胶时石油烃的回收率结果也列于表2。结果表明,除了无定型硅胶的回收率偏低外(<60%),球形硅胶的提取效率均达到70%~90%左右,但碱性硅胶回收率精密度不佳。故本工作选择使用中性球形硅胶进行各因素的考察。

**表 2:** 5种不同商品化硅胶的物化性质及其样品回收率

Source	Particle size/mesh	pH	Form	Specific surface area/ (m^2^/g)	Recoveries/ % (n=3)
Imported A	200-300	6-7	sphericity	490	87.5±5.0
Imported B	200-300	6-7	sphericity	480	68.7±3.2
Imported B	200-300	8-9	sphericity	480	69.7±15.8
Domestic A	200-300	6-7	amorphism	375	55.0±3.7
Domestic B	300-400	6-7	sphericity	370	75.4±1.5

### 2.4 硅胶用量的考察

由于土壤含水量是个变量,不能直接对硅胶绝对用量进行优化,本文对硅胶质量/(土壤样品质量×含水量)这个比例进行了优化。本文以10%含水率土壤为对象,分别加入实际含水质量1、2、5、10倍的硅胶进行研磨脱水,提取时间为20 min,回收率结果如图1。实验表明,加入硅胶的最佳方式是在研磨过程中,分次少量加入硅胶,随着硅胶的加入,土壤可以更快速流沙化。随着硅胶加入量的增加,石油烃的提取效率从61.8%逐渐提高到90%以上,呈上升趋势。硅胶的使用量对提取效率有较大的影响。当加入土壤实际含水质量10倍的硅胶时,回收率为92.9%,已满足要求。从原理上分析将硅胶用量增加到15~20倍可进一步增强除水效果及样品的分散性,回收率可能会更高。但对高含水量(≥15%)的样品,硅胶用量增加会导致提取时加入40.0 mL溶剂无法将全部样品浸泡在内,需要增加溶剂到50~60 mL,而这会显著提高检出限。综合考虑,本文最终选择加入10倍量的硅胶进行脱水。

**图 1:**
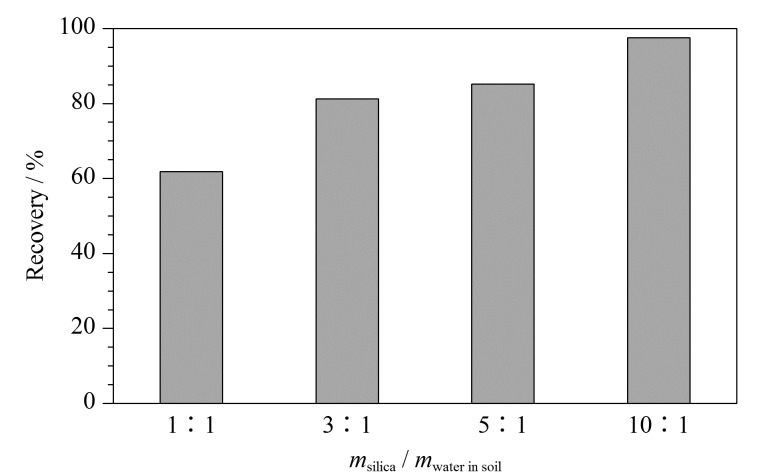
不同硅胶用量下土壤中石油烃的提取效率

### 2.5 提取方式及提取时长的考察

为满足现场快速检测的需要,减少现场操作的步骤,并同时满足红外光谱和气相色谱检测的需要,本研究以不含甲基的环己烷作为萃取溶剂,使用含水率10%的湿润土壤,对比了超声和摇匀后静置两种提取方式。分别考察了摇匀后静置提取5、10、15、20 min及超声提取5、10、15、20 min的石油烃提取效率,结果见图2。数据表明,超声提取的回收率在提取15 min时达到最大值;摇匀静置提取的回收率也在15 min达到最大(10 min为86.2% 15 min为88%)。经统计分析,超声提取与摇匀静置浸提对石油烃的提取效率无显著性差异(*P*>0.05)。从便捷性考虑,本研究选择了摇匀静置15 min。

**图 2:**
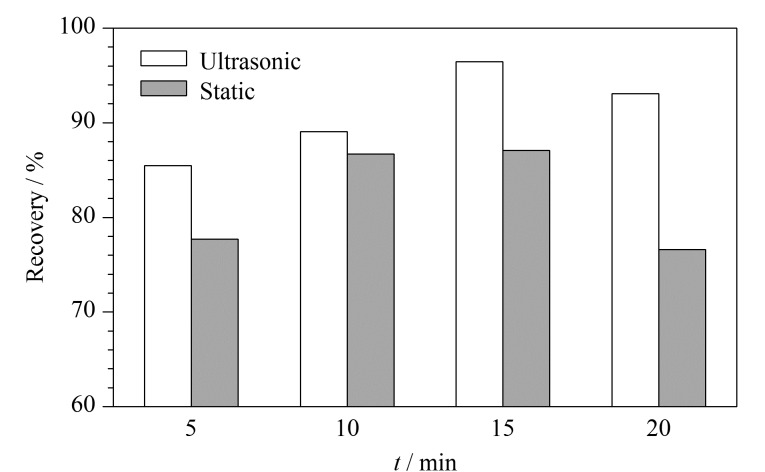
不同提取时间下土壤中石油烃的提取效率

现场检测是本法的主要应用场景。因为难以使用旋蒸设备,本法没有浓缩环节。虽然加大环己烷用量可进一步提高回收率,但同时也增大了稀释倍数降低了灵敏度,最终环己烷用量是保证回收率达到85%以上所需要的最小体积(10 g土壤40 mL溶剂)。这样做同时保证了灵敏度及回收率都能达到现场检测的要求。

### 2.6 方法学考察

#### 2.6.1 线性范围

用微量注射器(10、50、100 μL)分别移取10、25、50、100、200 μL的正构烷烃标准溶液,然后用移液枪分别加入9990、9975、9950、9900、9800 μL环己烷,配制成总石油烃质量浓度分别为310、775、1550、3100、6200 mg/L的标准工作液。原始正构烷烃标准中各正构烷烃质量浓度均为1000 mg/L,据此可以分别计算标准工作液中C10~C15、C15~C20、C20~C30、C30~C40各段的质量浓度。以此浓度与对应的色谱峰面积做线性回归,结果如表3所示,在各自的范围内,各碳数段均线性关系良好,相关系数(*R*^2^)均≥0.997。

**表 3:** 石油烃各组分的线性回归方程及其相关系数

Components	Linear range/(mg/L)	Regression equation	*R*^2^
C10-C15	60-1200	*Y*=3.29*X*+20.5	0.999
C15-C20	50-1000	*Y*=4.94*X*-29.7	0.999
C20-C30	100-2000	*Y*=3.49*X*+107.7	0.997
C30-C40	100-2000	*Y*=2.17*X*-6.4	0.998
C10-C40	310-6200	*Y*=3.45*X*-6.4	0.999

*Y*: total peak area of corresponding components; *X*: total mass concentration of corresponding components, mg/L; *R*^2^: correlation coefficient.

#### 2.6.2 定量限

由于石油烃的GC-FID谱图呈驼峰,因此根据仪器噪声计算检出限的方法不适用。参照现行环境标准HJ 1021-2019编制说明中^[19]^测定检出限及定量限的方法,在7份空白石英砂(10 g)中用移液枪添加400 μL混合石油烃溶液(加入量相当于400 mg/kg),按照本文方法进行测定。参照HJ 1021-2019编制说明以测量结果标准偏差(SD)的3倍确定检出限,定量限按照检出限的4倍确定。按照上述方式测得本方法的LOD为40 mg/kg, LOQ为160 mg/kg。

#### 2.6.3 回收率和精密度

采用本文的实验条件,取不同含量水平(200~1600 mg/kg)石油烃及不同含水率的土壤,加入土壤实际含水质量10倍的硅胶,以环己烷静态提取15 min后,经过Florisil固相萃取柱净化后检测,结果如表4。结果表明所有样品的回收率为74.1%~103.8%, RSD为1.7%~10.1%。表明本方法对不同浓度范围,不同含水量的样品,在不同碳数段上都具有良好的回收率和精密度。

**表 4:** 不同加标水平的石油烃在不同含水率土壤中的回收率及RSD(*n*=3)

Total content/ (mg/kg)	Soil moisture content/%	Recoveries/%(RSDs/%)				
		C10-C15	C15-C20	C20-C30	C30-C40	Total
1600	5	89.2 (8.8)	87.9 (8.1)	86.4 (10.1)	74.1 (7.3)	85.9 (9.0)
1600	10	85.6 (6.9)	82.6 (6.9)	88.8 (1.7)	91.9 (1.9)	87.7 (4.2)
1600	20	95.1 (5.2)	83.6 (6.7)	100.9 (5.2)	97.5 (6.7)	94.7 (4.5)
800	10	90.5 (5.6)	98.7 (4.7)	99.8 (6.8)	86.0 (1.6)	92.7 (5.1)
200	10	81.0 (4.6)	103.8 (6.0)	82.1 (6.9)	74.7 (5.9)	83.1 (5.2)

不同碳数的石油烃指纹是污染源判断的重要参考。石油烃在经过前处理后有可能造成不同碳数的石油烃回收率不同,为了保证指纹分析结果的准确性,要保证整个碳数范围都有良好的回收率。表4数据表明本文方法达到了这个要求。

### 2.7 实际样品分析

检测了盘锦地区某石油开采区附近两个土壤样品中石油烃的含量,结果见表5。结果表明,2个土壤样品中均检测出石油烃,其含量分别为1963 mg/kg和3419 mg/kg,均超过国家建设用地第一类用地筛选值(826 mg/kg),且不同样品之间石油烃的成分组成和比例相差较大。根据正构烷烃标准品保留时间及土壤样品的谱图(图3)推断,样品1主要由C15~C35时间段的石油烃组成,这是齿轮油的典型特征^[22]^;样品2主要由C10~C30时间段的石油烃组成,且呈现随保留时间的增加,正构烷烃含量逐次递减的规律,这说明该样品的主要污染物为原油^[22]^。上述判断与土壤样品的相关信息吻合。

**表 5:** 两个实际土壤样品中石油烃(C10~C40)的含量

Sample No.	C10-C15	C15-C20	C20-C30	C30-C40	Total
1	38	213	700	1012	1963
2	954	520	1692	253	3419

**图 3:**
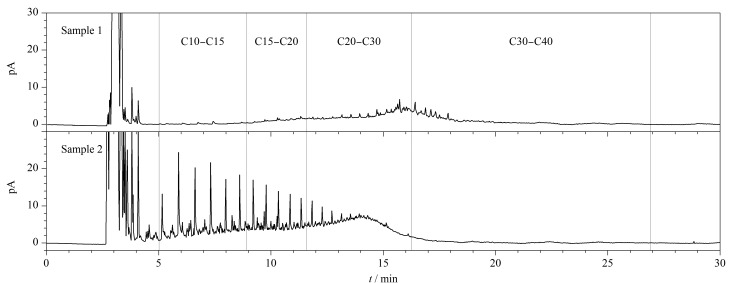
两个土壤样品中石油烃的气相色谱图

## 3 结论

本文提出了一种新的土壤中石油烃(C10~C40)的前处理方法。该方法将样品和硅胶充分混合研磨,利用硅胶脱除土壤中的水分,再以环己烷进行提取,Florisil固相萃取柱进行净化。以GC-FID对此方法的前处理效果进行了评价,结果表明该方法准确度高,精密度良好,回收率满意,能够有效提取、净化土壤中的石油烃(C10~C40)。本方法简便快捷,全程仅采用环己烷作为提取及净化溶剂,更具环保性。未来可以在红外光谱及便携气相色谱石油烃现场快速检测中发挥作用。由于缺少浓缩环节,本法灵敏度偏低。未来将研发简便灵活的现场样品浓缩设备,进一步改善本法的各项指标。
